# Bioactive Hexapeptide Reduced the Resistance of Ovarian Cancer Cells to DDP by Affecting HSF1/HSP70 Signaling Pathway

**DOI:** 10.7150/jca.62285

**Published:** 2021-08-24

**Authors:** Ruowen Guo, Qia Xu, Liwei Liu, Hui Liu, Yun Liu, Wenmei Wei, Yide Qin

**Affiliations:** 1 School of Basic Medical Sciences, Anhui Medical University, Hefei, Anhui 230032, P.R. China.; 2 Medical Laboratory Centre, PLA Clinical College (901 Hospital) of Anhui Medical University, Hefei, Anhui 230031, P.R. China.

**Keywords:** human ovarian cancer, drug resistance, bioactive hexapeptide, apoptosis, cell cycle, signaling pathway

## Abstract

Ovarian cancer is the leading cause of death in gynecologic malignancies. Ovarian cancer as a metastatic malignant tumor is highly recurrent and prone to drug resistance. Bioactive peptides are an emerging area of biomedical research in reducing resistance of tumor cell to drugs. In this paper, we investigated the effects and mechanisms of bioactive hexapeptide (PGPIPN) derived in milk protein on the sensitivity of ovarian cancer cells to cis-dichlorodiammine platinum (DDP). Human ovarian cancer cell lines (SKOV3 and COC1), their DDP-resistant sublines (SKOV3/DDP and COC1/DDP) and human primary ovarian cancer cells were cultured *in vitro* under the combined treatment of DDP (close to IC50) and different concentrations of PGPIPN. The viabilities, apoptosis and cell cycle changes were respectively measured by WST-8 and flow cytometry. The mRNA and protein expression levels of HSF1, HSP70, MDR1, ERCC1 and β-actin gene were respectively assayed by RT-qPCR and western blotting. The results showed that PGPIPN significantly increased the sensitivity of human ovarian cancer cells to DDP in inhibiting viability and inducing apoptosis *in vitro*. But the effects in sensitive cells were lower than DDP-resistant cells. PGPIPN significantly changed the cell cycles in all human ovarian cancer cells, which leaded to a significant increase in the percentage of cells blocked at G2/M phase and decrease the percentage of cells at G1 phases in a dose-dependent manner. PGPIPN affected the expression levels of HSF1, HSP70, MDR1 and ERCC1 genes. Compared with cells in DDP treatment alone, the expression levels of HSF1 and HSP70 in human ovarian cancer cells treated with DDP and PGPIPN together significantly decreased in dose-dependent manner. PGPIPN significantly decreased MDR1 and ERCC1 of drug-resistant ovarian cancer cell lines and human primary ovarian cancer cell in a dose-dependent manner. Pifithrin-μ (PFTμ, HSP70 inhibitor) decreased or removed the effects of peptide in increasing the sensitivity of ovarian cancer cells to DDP. This suggests that PGPIPN enhanced the sensitivity of ovarian cancer cells to DDP partially via reducing the activity of HSF1/HSP70 signaling pathway, thus inducing cell apoptosis and decreasing repairment of DNA damage.

## Introduction

Ovarian cancer is a gynecological malignant tumor with high mortality 1, 2. Early ovarian cancer is not easily diagnosed because it is concealed and there is not a landmark clinical indicator 3. Ovarian cancer is prone to metastasis. Once metastasis occurs, chemotherapy is mainly treatment to prolong the survival of patients. However, chemotherapy is prone to drug resistance and the therapeutic effect is gradually reduced, resulting in high patient mortality 4-6. Relevant statistics showed that ovarian cancer patients who respond to chemotherapy initially all built drug resistance due to the long-term use of chemotherapy drugs 7-10. Drug resistance reduced the effective treatment of patients, and thus the 5-year survival rate of patients with advanced ovarian cancer is lower than 30% 11. Therefore, how to enhance the sensitivity of ovarian cancer to chemotherapy drugs has gradually become a new research direction.

Current first-line treatment of high-grade epithelial ovarian cancer includes debulking surgery followed by combination chemotherapy, usually platinum and paclitaxel 12. Platinum mainly includes carboplatin and cisplatin/cis-diaminodichloroplatinum (DDP or CDDP). DDP is a common chemotherapeutic drug for the treatment of ovarian cancer, which is widely used in the world due to its broad anticancer effect, its synergistic effect with other antitumor drugs, and its lack of cross-resistance 13, 14. But the use of DDP is associated with drug resistance, limiting the clinical application of DDP. The resistance of DDP mainly refers to the fact that the patient does not achieve the therapeutic effect due to repeated use of the initially metered DDP and the dose must be increased. Related studies have shown that DDP resistance mainly has three molecular mechanisms: increased DNA repair, altered drug accumulation in cells and increased drug inactivation 15, 16. The studies in recent years have found a variety of drugs that can reverse tumor multidrug resistance *in vitro*, but most these drugs have the defect of cytotoxicity, which limits their clinical application 13. Therefore, the development of new drugs with low toxicity and effective reversal of tumor resistance has become more and more urgent.

In recent years, milk-derived bioactive peptides have become a focus of anti-cancer medical research. Some studies have found that milk-derived bioactive peptides have an important role in immune regulation and anti-tumor 17, 18. Studies have also found that some bioactive peptides also play a role in enhancing the sensitivity of cancer cells to chemotherapeutic drugs and reversing the resistance of cancer cells 19, 20. Many research scholars believe that some milk-derived bioactive peptides are a natural reversal agent to reverse cell resistance, but the research has just started and there are few reports on related research. Pro-Gly-Pro-Ile-Pro-Asn (PGPIPN) is a hexapeptide derived from the 63-68 amino acid residue of bovine β-casein 21. We found in previous experiments that PGPIPN could effectively reduce inflammation and promote the immune of body, thereby reducing alcoholic liver injury 22, 23. It also can inhibit the proliferation, invasion and metastasis of ovarian cancer cells and induce the cells apoptosis 18, 24, 25. At the same time, our research indicated that PGPIPN had no toxic side effects on normal cells (non-transformed cells), but its anticancer efficiency was inferior to traditional chemotherapy drugs, such as DDP. Our latest study showed that the combination of PGPIPN and DDP can significantly improve the DDP effect on anti-cancer. In this study, we evaluated whether PGPIPN can enhance the sensitivity of human ovarian cancer to DDP (proliferation, apoptosis and cell cycle), and explored the possible mechanism of this peptide to sensitize ovarian cancer cells in human ovarian cancer cell lines (SKOV3 and COC1), DDP-resistant SKOV3 (SKOV3/DDP), DDP-resistant COC1 (COC1/DDP), human primary ovarian cancer cell and human primary normal ovarian cell. The purpose of our study was to reduce the adverse reactions of patients to chemotherapy drugs, and finally provide a new treatment for drug-resistant ovarian cancer.

## Materials and methods

### Reagents

PGPIPN (purity >99.5%, confirmed by reversed phase high performance liquid chromatography) was supplied by Sangon Biotech Co., Ltd. Hematoxylin solution (catalog #: MHS1) and eosin (catalog #: 861006) were purchased from Sigma‑Aldrich, Merck KgaA, Germany. Rabbit polyclonal or monoclonal antibodies of human HSF1 (catalog #:ab52757), Hsp70 (catalog #:ab181606), MDR1 (P-glycoprotein, P-gp; catalog #:ab170904), ERCC1 (catalog #: ab129267), cytokeratin 7 (catalog #: ab181598), cytokeratin 19 (catalog #: ab52625) and β-actin (catalog #: ab8227) were purchased from Abcam, USA. Secondary antibody (horseradish peroxidase‑conjugated goat anti‑rabbit IgG, catalog #: GAR‑HRP) and Super Signal West Pico kit (ECL Chromogenic kit) were purchased from Thermo Fisher Scientific, Inc. Secondary antibody (FITC-labeled Goat Anti-Rabbit IgG (H+L), catalog #: A0562) and Hoechst 33342 (catalog #: C1022) were Beyotime Biotechnology Co. Ltd., Shanghai, China. Pifithrin-μ (PFTμ, HSP70 inhibitor, catalog #: ab1202886) was purchased from Abcam, USA.

### Cell culture

Human ovarian cancer cell line SKOV3 and COC1 were originally purchased from ATCC (American Type Culture Collection, USA) and preserved by the Biochemistry and Molecular Biology Laboratory of Anhui Medical University. DDP-resistant human ovarian cancer cell line SKOV3/DDP and COC1/DDP were purchased from Shanghai Ran Biotechnology Co., Ltd., China. SKOV3 and SKOV3/DDP were cultured in McCoy's 5A (Modified) medium, and cell line COC1 and COC1/DDP were cultured in RPMI-1640 medium, all supplemented with heat-inactivated 10% FBS (Fetal Bovine Serum), penicillin (100 U/ml) in 5% CO_2_ at 37 °C. After the cells grow in the logarithmic phase were digested with 0.4% trypsin, centrifuged, and changed the medium every other day. The drug-resistant strain cell lines were resistant to DDP and were prepared by repeated and intermittent treatment with 0.3 μg/ml DDP.

Fresh primary ovarian tumor tissues classified as high grade serous carcinoma (HGSC, III-IV grade) according to WHO criteria were collected from 6 ovarian cancer patients with progressive disease/stable disease (PD/SD) who underwent debulking surgery at the first affiliated hospital of Anhui Medical University between January 2017 and December 2018. These patients have received DDP or carboplatin chemotherapy. For comparison, normal ovarian glandular epithelium cells (NOGECs) were cultured from fresh primary normal ovarian tissues harvested from 6 patients with uterine fibromas, which were confirmed as negative for any neoplastic disease by pathological examination. Prior to tissue deposition, all patients signed written consent forms confirming their donation of tissue for research purposes according to the Declaration of Helsinki. This study was approved by the Biomedical Ethics Committee of Anhui Medical University (approval number: 20170213). Fresh primary ovarian tumor tissues were slowly washed with PBS 2-3 times, the tumor tissue was cut into small pieces about 1 mm^3^, transferred to 50 ml sterile centrifuge tube with 0.4% trypsin and digested at 37 °C for 40-50 min. Then the tumor tissue fragment was centrifuged at 800 rpm for 5 min, and the cell pellet was resuspended in RPMI-1640 medium with 10% fetal bovine serum (FBS) containing 0.1 mg/l epidermal growth factor (EGF), 0.1 mg/l insulin-like growth factor (IGF) and 0.1 mg/l β-estradiol, then cultured in 5% CO_2_ at 37 °C. After 48 h, we washed the unattached tissue with PBS, and the adherent cells were further cultured until the confluence rate was 70-80 %, and then digested with 0.4% trypsin for purity identification. To determine purity, cells were analyzed using immunofluorescence of cytokeratin 7 (for ovarian cancer cells) or cytokeratin 19 (for normal ovarian epithelium cells).

### H&E stained

The cells in the logarithmic growth were pretreated with different drug groups and put on sterilized slides at the bottom of the 6 well plates. Then put in 37 °C, 5% CO_2_ incubator for 48 h. The slides were removed from the plates, fixed, and the cells were stained with hematoxylin and eosin. Next, they were washed with water and oven-dried. The slides were sealed with a neutral resin, observed under an upright microscope and photographed. Fresh ovarian normal and tumor tissues and were fixed with 4% paraformaldehyde solution at room temperature for 24 h, dehydrated with alcohol, embedded in paraffin and prepared into tissue sections. These tissues stained with hematoxylin and eosin (H&E) were observed and analyzed under an optical microscope at ×100 magnification.

### Cell viability assay

The logarithmically grown cells were seeded into 96-well plates in sextuplicate at a starting density of 5 × 10^3^ cells/well, and incubated with the medium containing various drugs (0 as control, 25 μmol/L (μM) DDP, 25 μM DDP+0.15 μM PGPIPN, 25 μM DDP+1.5 μM PGPIPN and 25 μM DDP+15 μM PGPIPN, respectively). After cultured for 24, 48 and 72 h, these cells were incubated with WST-8 (water-soluble tetrazolium 8) solution by Cell Counting Kit-8 (CCK8) for 4 h at 37 °C and 5% CO_2_. The cells were rocked on a shaker for 2 min; the supernatants were collected and measured at 450 nm.

In addition, the above cell lines (SKOV3, SKOV3/DDP, COC1 and COC1/DDP) were also seeded into 96-well plates respectively in sextuplicate at a starting density of 5 × 10^3^ cells/well, and incubated with the medium containing various drugs (0 as control, DDP, DDP+PGPIPN, DDP+PFTμ, DDP+PGPIPN+PFTμ and PFTμ respectively) for 48 h, in which the concentrations of DDP, PGPIPN and PFTμ were respectively 25, 1.5 and 20 μM. The assays of cell viability were as aforementioned mathod.

The percent viability of cells was calculated using the following formula. Viability (%) = (the experimental group A_450 nm_ /control group A_450 nm_) × 100%. Each experiment was triplicated independently. The experiments were duplicated with primary ovarian cancer and normal cells from six patients, respectively.

### Flow Cytometry Analysis

After 48 h of different drugs (0, 25 μM DDP, 25 μM DDP+0.15 μM PGPIPN, 25 μM DDP+1.5 μM PGPIPN and 25 μM DDP+15 μM PGPIPN, respectively) incubations in 6-well culture plates, the cells were digested with trypsin without EDTA and washed twice with pre-cooled PBS. The apoptosis of cells was measured by flow cytometry (FCM) using FITC-conjugated Annexin-V and propidium iodide (PI) from Apoptosis Detection Kit (Bestbio, Shanghai) according to the manufacturer's instructions. The cycle of cell stained with PI was measured by FCM from Cell Cycle Detection Kit (Bestbio, Shanghai) according to the manufacturer's instructions. For cell cycle distribution analysis, per sample analyzed 10,000 cells. The analysis of cell apoptosis and cycle was performed using Flowjo 7.6.1 software. The experiments in cell lines were performed with triplicate, and the experiments were duplicated with primary ovarian cancer cells from six patients.

### Reverse transcription-quantitative PCR (RT-qPCR)

The primers were designed by Primer 6.0 software according to genes sequences searched by Primer-Bank, and synthetized by Shanghai Shenggong Biological Co., Ltd. (Table 1). The ovarian cancer cells were harvested after different drug (the drug administrations and concentrations were consistent with the aforementioned flow cytometry) treatments for 48 h, in which the total RNAs were extracted with TRIzol transcription kit (cat. no. 15596026; Invitrogen; Thermo Fisher Scientific, Inc.) according to the manufacturer's instructions. RNA was reverse-transcribed to cDNA using TRIzol transcription kit (Thermo Scientific) following the manufacturer's instructions. The reverse transcription reaction conditions were 42 °C for 1 h and 70 °C for 5 min. Real time PCR adopts TaKaRa SYBR Green as real time PCR Master Mix in ABI7500 fluorescent real-time PCR instrument (Applied Biosystems; Thermo Fisher Scientific, Inc.). The reaction conditions were as follows: 95 °C pre-denaturation 300 s; 95 °C ×20 s, 56 °C×30 s, 72 °C×30 s (40 cycles). β‑actin was used as the housekeeping gene. For each target gene, mRNA expression levels were calculated using the 2^‑ΔΔCq^ method (ΔCq=target gene Cq ‑ β‑actin Cq value). The experiments in cell lines were performed with triplicate in two independent sets, and the experiments were duplicated with primary ovarian cancer cells from six patients. And a mixture lacking a complementary DNA template was used as the negative control.

### Western blotting

The ovarian cancer cells under were collected and harvested after different drug (the drug administrations and concentrations were consistent with the aforementioned flow cytometry) treatments for 48 h, in which the cell proteins were extracted and the proteins of the above cells were separated by SDS-PAGE, and transfer to PVDF membrane. After blocking with 5% milk, the membranes were incubated with primary antibodies (1:1,000) and then incubated with horseradish peroxidase conjugated secondary antibody (HRP‑conjugated goat anti‑rabbit IgG, 1:8,000) overnight. Proteins were detected using the ECL system and exposed in a chemiluminescent imaging system (Clinx Science Instruments Co., Ltd.), and intensities were quantified using Quantity-One software version 4.62 (Bio-Rad). The β-actin was used as a loading control. The experiments in cell lines were performed with triplicate in two independent sets, and the experiments were duplicated with primary ovarian cancer cells from six patients.

### Statistical analysis

All statistical analyses were performed using SPSS 20.0 software (IBM Corp.). Data are presented in mean ± *SD*. The comparisons between the treatments and control were analyzed with one-way ANOVA followed by Tukey's post hoc test. Results were considered statistically significant at *P*<0.05.

## Results

### Culture and identification of primary cells

**Figure 1:**
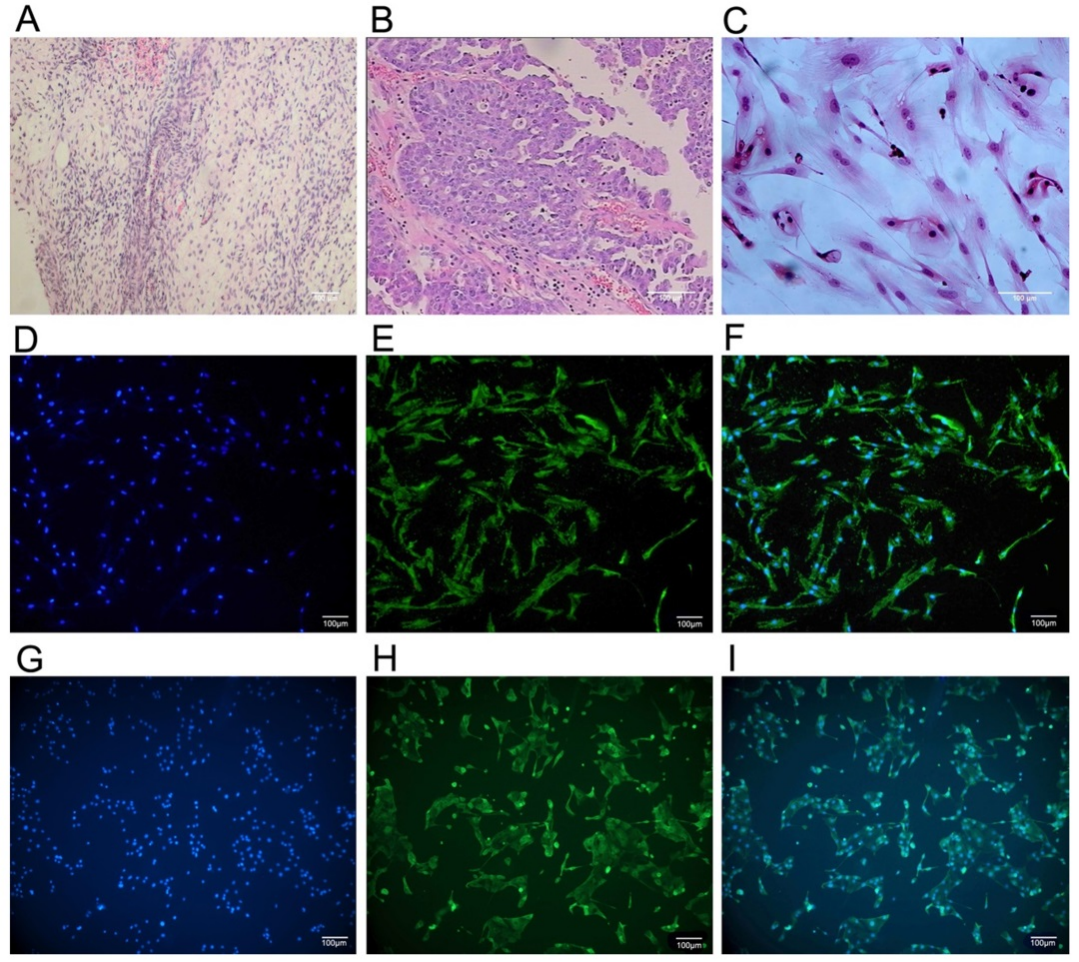
** The culture and identification of human primary ovarian cancer cells. (A)** Pathological section of normal human ovarian tissue with benign pathology (H&E stained, ×40). **(B)** Pathological section of human ovarian cancer tissue (H&E stained, ×100) that was classified as serous ovarian adenocarcinoma (IV grade) according to WHO criteria. **(C)** Human primary ovarian cancer cells subcultured in medium (H&E stained, ×200). **(D)** Cultured human primary normal ovarian glandular epithelium cells (NOGECs) stained with nuclear dyes-Hochest 33258 (×100). **(E)** Cultured human primary NOGECs stained with anti-cytokeratin 19-FITC (×100). **(F)** The confocal of D and E pictures (×100). **(G)** Cultured human primary ovarian cancer cells stained with nuclear dyes-Hochest 33258 (×100). **(H)** Cultured human primary ovarian cancer cells stained with anti-cytokeratin 7-FITC (×100). **(I)** The confocal of G and H pictures (×100).

The primary ovarian cancer cell lines were successfully isolated and established from 6 ovarian cancer patients who underwent debulking surgery in the first affiliated hospital of Anhui Medical University. Immunocytochemistry analysis of anti-cytokeratin 7 staining (Figure 1) revealed the more than 90% purity of ovarian cancer cell within the isolated cell populations. For comparison, we also isolated and established normal ovarian glandular epithelium cells (NOGECs) lines from 6 patients with uterine fibromas, which were confirmed as negative for any neoplastic disease by pathological examination. The anti-cytokeratin 19 was used for immunocytochemistry analysis of NOGECs, in which purity of NOGECs was about 80.95% within the isolated cell populations (Figure 1).

### PGPIPN enhanced DDP inhibition of human ovarian cancer cell viability

**Figure 2:**
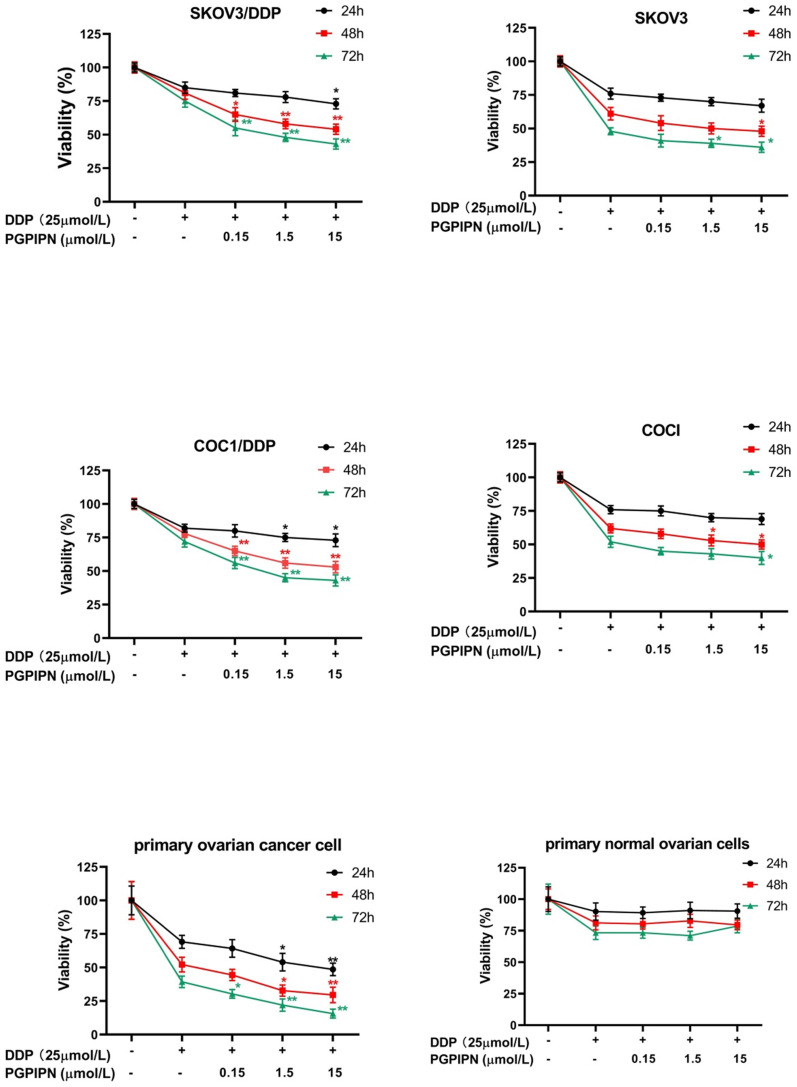
** PGPIPN increased the sensitivity of human ovarian cancer cells to DDP in the inhibition of cell viability* in vitro*, of which the effect of resistant cells were better than that of sensitive cells.** Data are mean ± *SD*, the experiment in cell lines were performed in triplicate, and the experiments were duplicated with primary ovarian cancer cells from six patients. ^*^P<0.05, ^**^P<0.01 compared with human ovarian cancer cell lines, human primary ovarian cancer cells or human primary normal ovarian cells intervened by DDP alone in the same time.

Compared with cells in DDP treatment alone, the viabilities of drug-resistant ovarian cancer cells (SKOV3/DDP and COC1/DDP) treated with DDP and PGPIPN together significantly decreased in dose-dependent manner (*P*<0.05 or *P*<0.01). But the effects in sensitive cells (SKOV3 and COC1) were not obvious (Figure 2). Compared with cells treated with in DDP treatment alone, treatment of primary ovarian cancer cells with DDP and PGPIPN together led to a significant dose-dependent decrease in cell viability, of which effects were better than that in human ovarian cancer line cells (*P*<0.05 or *P*<0.01, Figure 2). However, the above treatment had no effect on non-transformed cells (NOGECs, Figure 2). These results indicated that PGPIPN reduced the resistance of ovarian cancer cells to DDP.

### PGPIPN increased DDP-induced apoptosis in human ovarian cancer cells

**Figure 3:**
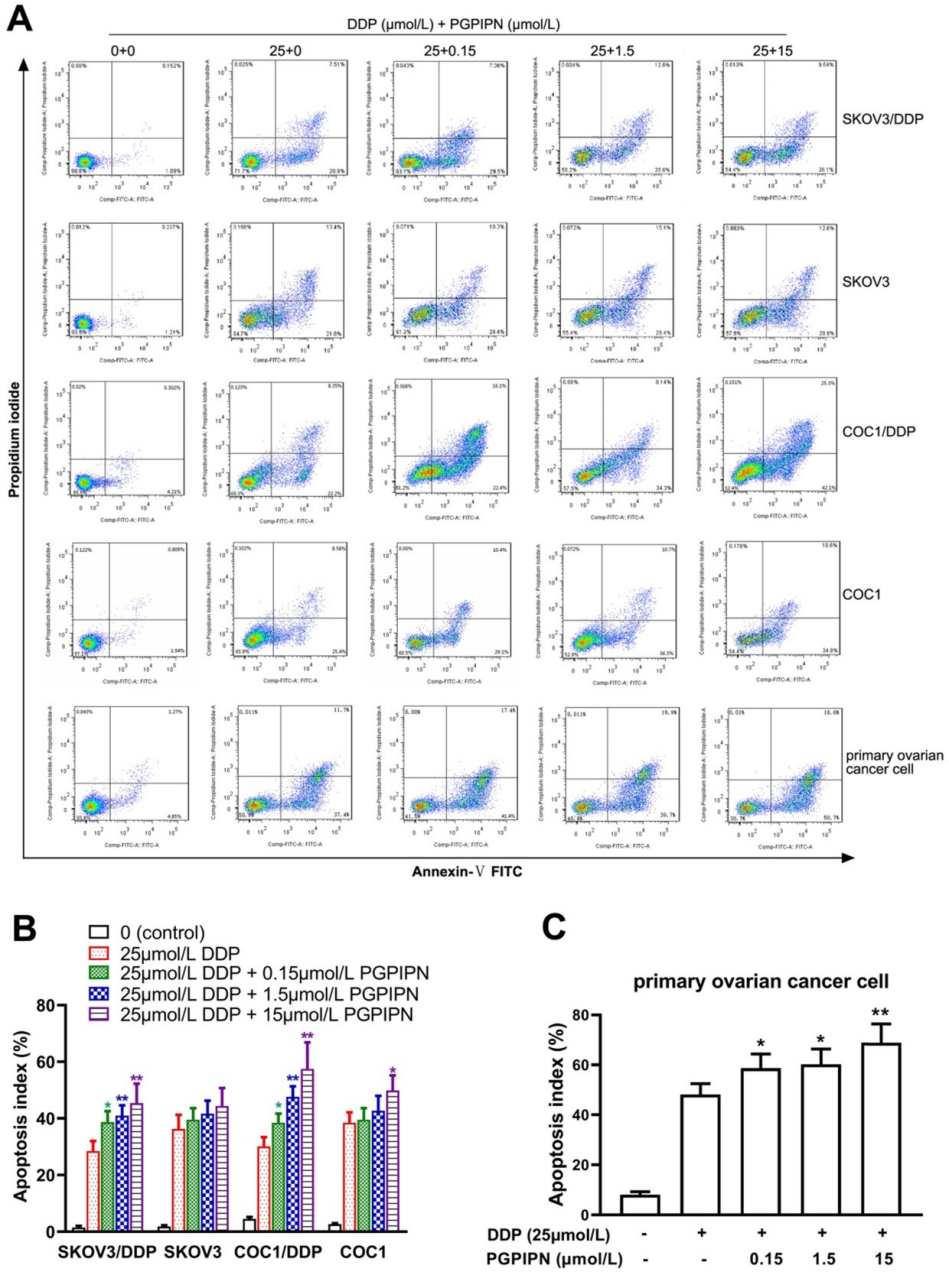
** PGPIPN increased DDP-induced apoptosis in human ovarian cancer cells for 48 h *in vitro*. (A)** Representative flow cytometry dot plot of human ovarian cancer cells stained with Annexin-V-FITC and PI. **(B)** Histogram of apoptosis rates of lines (SKOV3, COC1) and their DDP-resistant sublines (SKOV3/DDP, COC1/DDP). **(C)** Histogram of apoptosis rates of human primary ovarian cancer cell. The data are shown as means ± SD, the experiment in cell lines was performed in triplicate, and the experiments were duplicated with primary ovarian cancer cells from six patients, ^*^P<0.05, ^**^P<0.01 compared with cells in DDP treatment alone.

According to analysis of flow cytometry using an annexin V-TITC and PI double-staining method, PGPIPN could significantly promote the apoptosis of resistant cell lines (SKOV3/DDP and COC1/DDP) and human primary ovarian cancer cells inducing by DDP for 48 h, which displayed dose-dependent manners (Figure 3). Compared with cells in DDP treatment alone, the apoptosis rates of drug-resistant ovarian cancer cells (SKOV3/DDP and COC1/DDP) and human primary ovarian cancer cells treated with DDP and PGPIPN together significantly increased (*P*<0.05 or *P*<0.01). However, the effects in sensitive cells (SKOV3 and COC1) were not obvious except for large doses of peptides (Figure 3). This indicates that PGPPN has the effect of promoting DDP-induced apoptosis of ovarian cancer cells.

### PGPIPN enhanced DDP effect on cell cycle of human ovarian cancer

**Figure 4:**
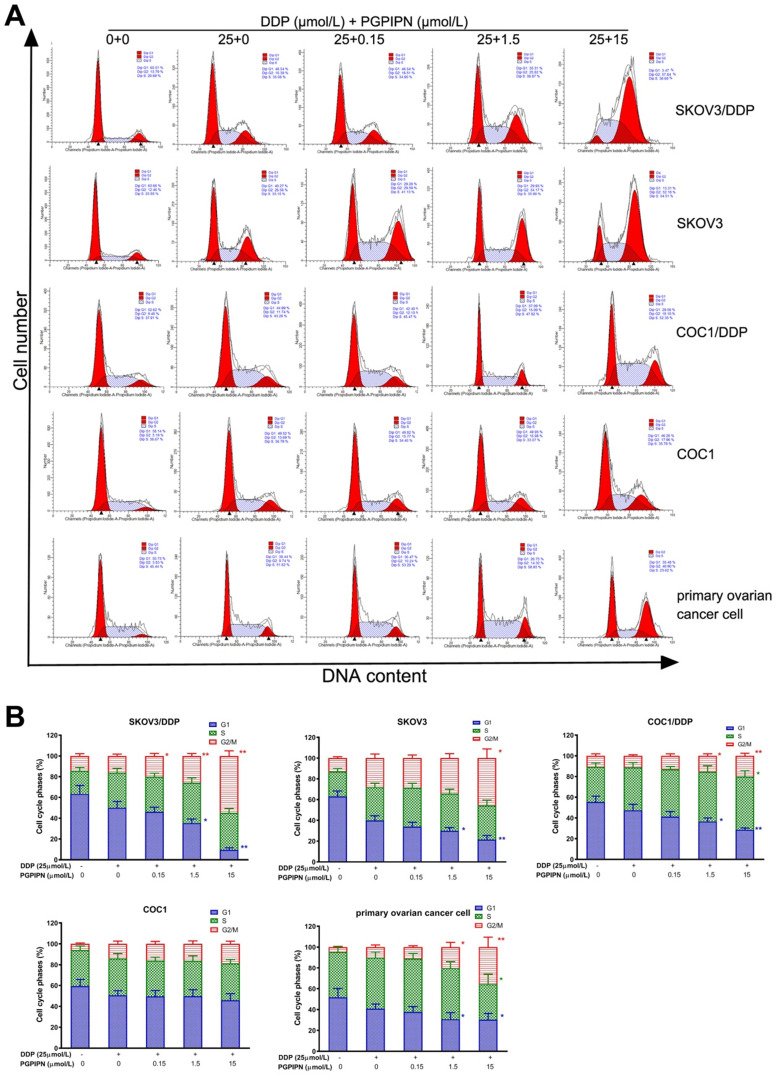
** PGPIPN induced G2/M-phase accumulation of human ovarian cancer cells under combining drugs for 48 h. (A)** Representative cell cycle profiles by flow cytometry. **(B)** The histograms were analyzed by Flowjo software to display the cell cycle distribution. The data are shown as means ± SD, the experiment in cell lines was performed in triplicate, and the experiments were duplicated with primary ovarian cancer cells from six patients, ^*^P<0.05, ^**^P<0.01 compared with cells in DDP treatment alone.

To investigate the effect of combination (DDP+PGPIPN) on cell cycle progression, we performed flow cytometry analysis and examined the synergistic effect on G1, S and G2/M phase within the cell cycle in SKOV3, SKOV3/DDP, COC1 and COC1/DDP human ovarian cancer cell lines, and human primary ovarian cancer cell. The results showed that the cell cycles were significantly changed under combining drugs for 48 h in all human ovarian cancer cells. The results also showed PGPIPN resulted in a marked increase in the percentage of cells blocked at G2/M phase and significantly reduced the percentage of cells at G1 phases, which showed a dose-dependent manner (Figure 4). These results suggest that the combination of DDP and PGPIPN causes the highest number of cells arrested at G2/M phase compared to DDP alone in both human ovarian cancer cell lines and primary ovarian cancer cell (*P*<0.05 or *P*<0.01). This indicates that PGPPN has the effect of promoting DDP-induced G2/M arrest of ovarian cancer cell cycle.

### PGPIPN regulates the expression of genes associated with HSF1/HSP70 signaling pathway in enhancing the effect of DDP

**Figure 5:**
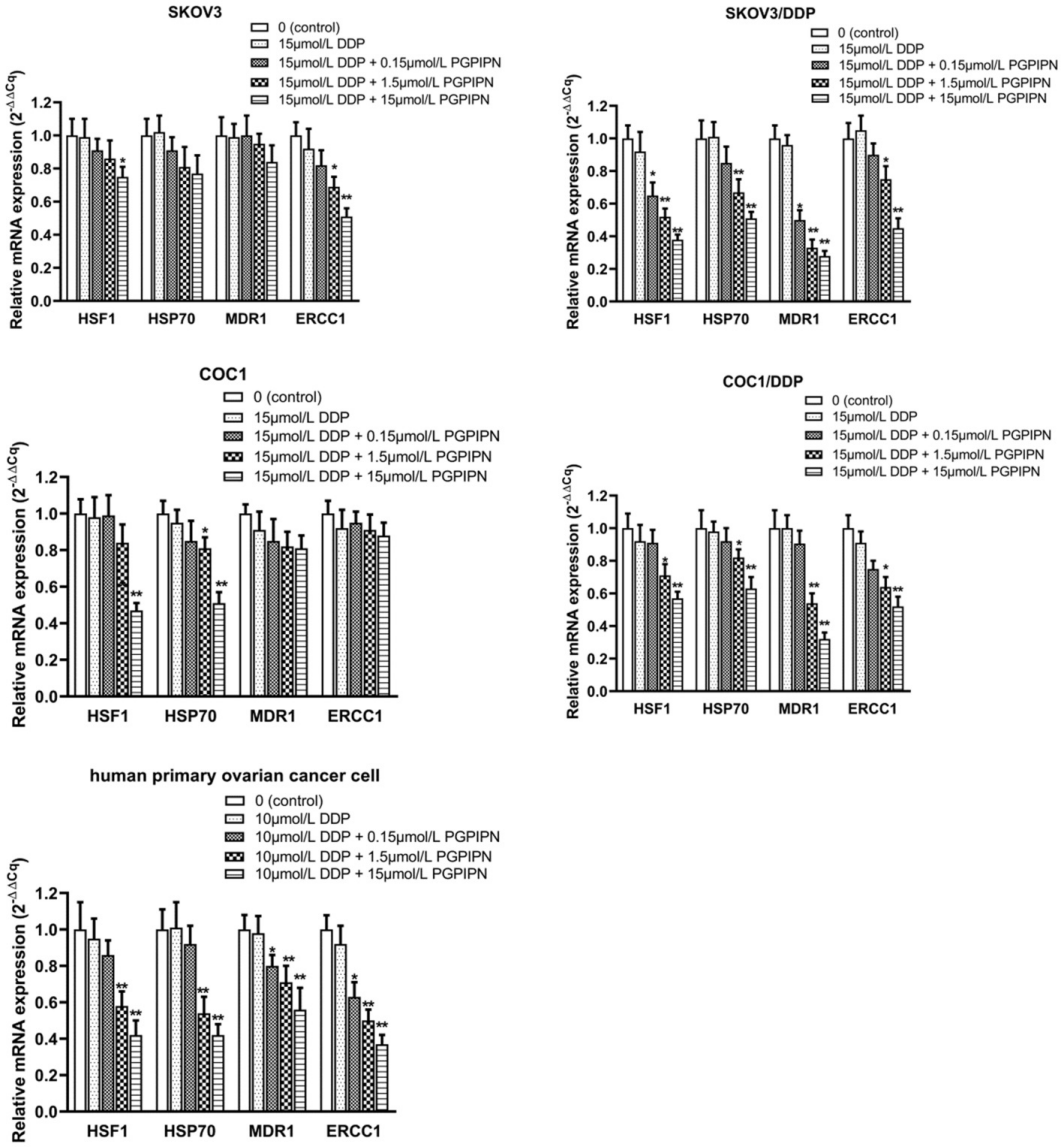
** PGPIPN regulates the mRNA expression levels of HSF1, HSP70, MDR1 and ERCC1.** β‑actin was used as the reference gene. Data are presented as the mean ± SD (cell line: n=6; primary ovarian cancer cell: n=12). ^*^P<0.05 and ^**^P<0.01 vs. human ovarian cancer cells in DDP treatment alone.

This study examined the mRNA expression levels of HSF1, HSP70, MDR1 and ERCC1 by RT‑qPCR in SKOV3, SKOV3/DDP, COC1, COC1/DDP and human primary ovarian cancer cells, of which MDR1 and ERCC1 are important drug-resistance genes. Compared with cells in DDP treatment alone, PGPIPN significantly reduced the mRNA level of HSF1 and HSP70 in both human ovarian cancer cell lines and primary ovarian cancer cell, which displayed a dose-dependent, and the effect of drug-resistant cells (SKOV3/DDP, COC1/DDP) were significantly better than that of sensitive cells (SKOV3, COC1) (Figure 5). Similarly, PGPIPN significantly reduced MDR1 and ERCC1 mRNAs of drug-resistant ovarian cancer cell lines and human primary ovarian cancer cell in a dose-dependent manner. However, the mRNA expression of sensitive cells (SKOV3, COC1) did not change significantly in PGPIPN treading, except for SKOV3 cells at high doses of the peptide (Figure 5). Overall, PGPIPN could reduce the mRNA level of genes associated with HSF1/HSP70 signaling pathways in drug-resistant ovarian cancer cell lines and human primary ovarian cancer cell.

**Figure 6:**
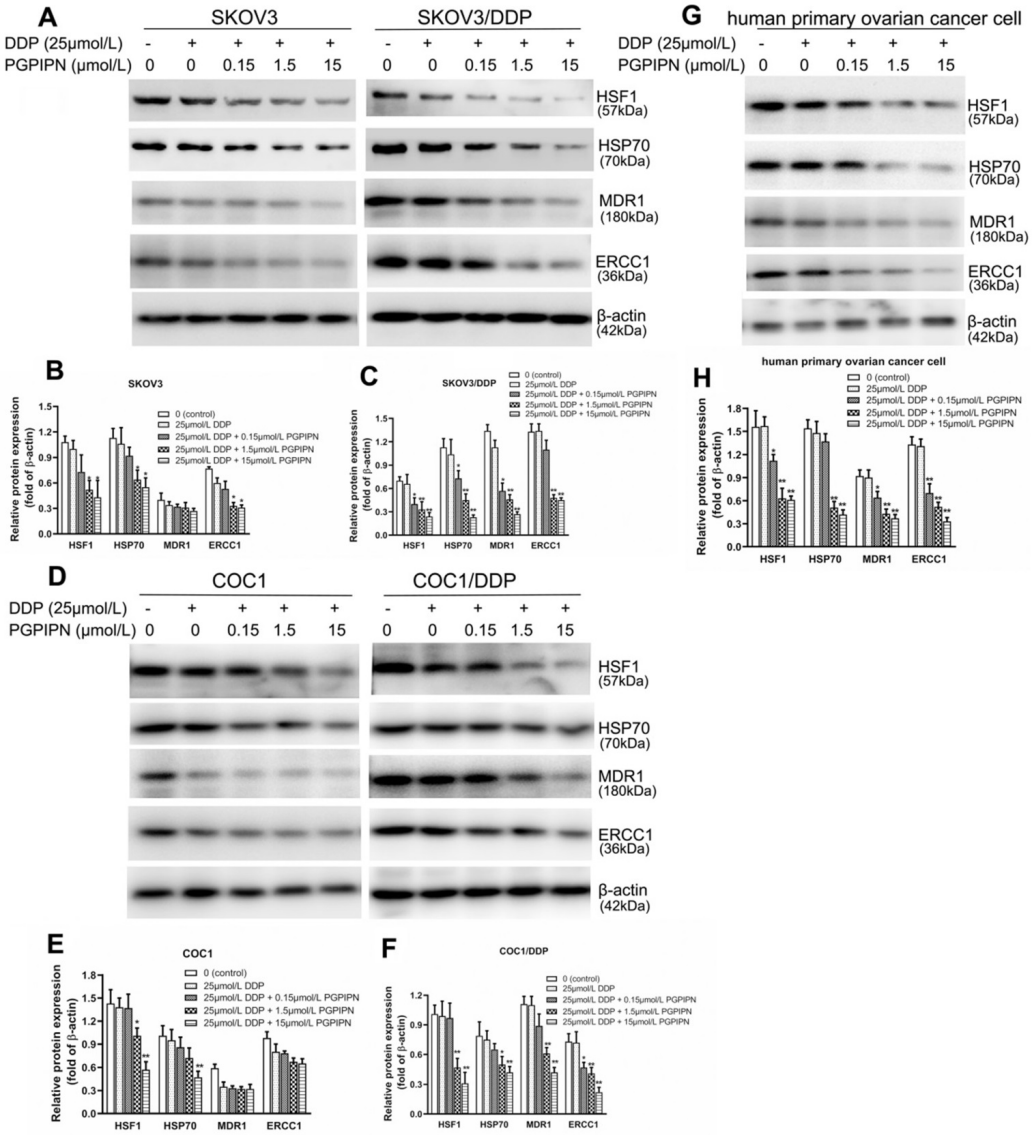
** PGPIPN regulated the contents of proteins associated with HSF1/HSP70 signaling pathway in human ovarian cancer cell lines and primary ovarian cancer cells.SKOV3 and its DDP-resistant subline SKOV3/DDP. (A)** The related proteins were detected with western blotting in SKOV3 and SKOV3/DDP, and β-actin was used to show the similar amount of protein loaded in different lanes. **(D)** The related proteins were detected with western blotting in COC1 and COC1/DDP, and β-actin was used as the reference protein. **(G)**The related proteins were detected with western blotting in human primary ovarian cancer cells, and β-actin was used as the reference protein.** (B, C, E, F and H)** Relative intensities of protein bands in A, D and G were determined using Quantity-One software and normalized using β-actin band intensity. Data in B, C, E, F and H are presented as mean ± *SD* (cell line: n=6; primary ovarian cancer cell: n=12). ^*^P<0.05, ^**^P<0.01 vs. cells in DDP treatment alone.

Western blotting was used to analyze HSF1, HSP70, MDR1 and ERCC1 proteins, which are related to HSF1/HSP70 signaling pathway in SKOV3, SKOV3/DDP, COC1, COC1/DDP and human primary ovarian cancer cells. Compared with cells in DDP treatment alone, the protein expression levels of HSF1 and HSP70 in human ovarian cancer cells treated with DDP and PGPIPN together significantly decreased in dose-dependent manner (*P*<0.05 or *P*<0.01, see in Figure 6). PGPIPN significantly decreased MDR1 and ERCC1 proteins of drug-resistant ovarian cancer cell lines and human primary ovarian cancer cell in a dose-dependent manner (*P*<0.05 or* P*<0.01, see in Figure 6). MDR1 and ERCC1 proteins were low in the drug-sensitive cell lines (SKOV3/DDP, COC1/DDP), and PGPIPN had little effect on these two protein expression levels, although the peptide at moderate- and high doses could affect ERCC1 protein expression levels in SKOV3 cell (see in Figure 6). Consequently, PGPIPN had the most significant effects on the HSF1/HSP70 pathway, which resulted in reduced protein MDR1 and ERCC1 associated with drug-resistance especially in resistant strain cells.

**Figure 7:**
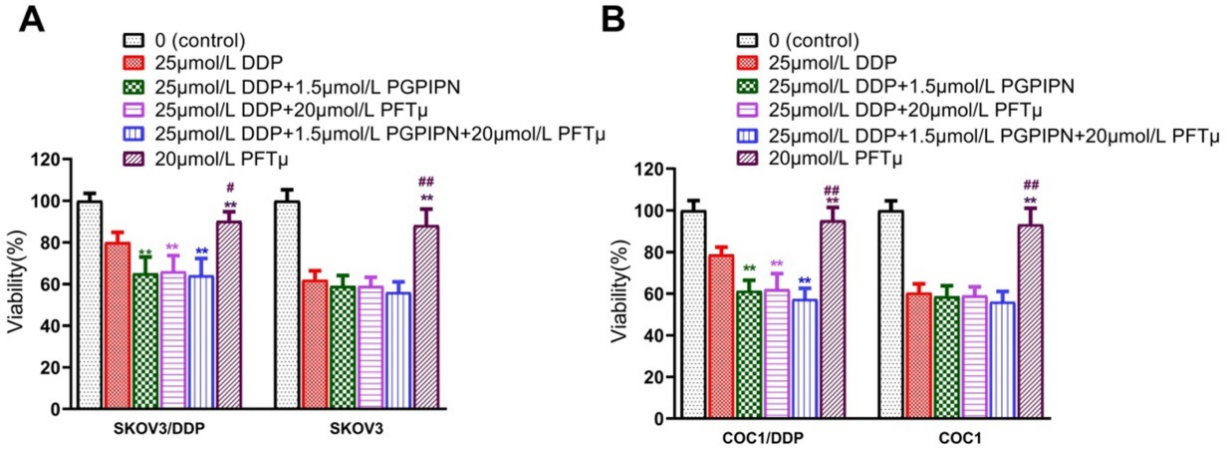
** HSF1/HSP70 signaling pathway inhibitors reduced the effect of PGPIPN. (A)** SKOV3 and DDP-resistant subline SKOV3/DDP. **(B)** COC1 and DDP-resistant subline COC1/DDP. Data are mean ± SD, the experiment was performed with sextuplicate in three independent sets, ^*^P<0.05, ^**^P<0.01 compared with DDP intervention alone in the same cell line; ^#^*P*<0.05, ^##^*P*<0.01 compared with DDP and PGPIPN synergistic intervention in the same cell line.

To verify the result that PGPIPN regulates HSF1/HSP70 signaling pathway in enhancing the effect of DDP, we conducted the experiment of inhibiting HSP70. The results suggested that once the HSF1/HSP70 signaling pathway was inhibited by HSP70 inhibitor (PFTμ), PGPIPN did not further inhibit the cell activities. Since HSF1/HSP70 pathway had been downregulated by PFTμ, and there was not further potential for the peptide to suppress this pathway (Figure 7). So HSP70 inhibitor (PFTμ) replaced or removed the effects of the peptide in increasing the sensitivity of ovarian cancer cells to DDP, i.e. PFTμ replaced the partial function of the peptide (Figure 7). This suggested that PGPIPN may regulate HSF1/HSP70 signaling pathway activity and following cascade to enhance the sensitivity of ovarian cancer cells to DDP.

## Discussion

At present, the main chemotherapeutic drugs for ovarian cancer are cisplatin, paclitaxel and so on^26^. However, due to the emergence of drug resistance in ovarian cancer, the therapeutic effect of cisplatin is less potent, and there is still the high mortality of ovarian cancer 27. There is an urgent need for a new drug in clinical practice to improve the results of chemotherapy, reduce the resistance of chemotherapy, and improve the survival rate of patients. Existing drugs that improve the sensitivity of patients to chemotherapy include calcium blockers (verapamil), antisense sequences of resistance-related genes, oligonucleotides, hormones (progestins), and so forth 28, 29. The problem with these drugs all has toxic side effects, such as cardiovascular and renal toxicity, making it not widely used clinically. Therefore, the discovery of a new non-toxic or low-toxic reversal drug is urgently needed.

Bioactive peptides have achieved certain results in the treatment of tumors and the sensitization of chemotherapeutic drugs 30-32. In this research, we used milk-derived hexapeptide (PGPIPN), which is a product of bovine casein proteolysis. The results of this experiment indicate that PGPPN can enhance the effect of cisplatin on ovarian cancer cells and enhance the sensitivity of ovarian cancer to cisplatin. According to our previous experiments, PGPIPN also inhibited cell growth alone, but the peptide inhibited the viability of ovarian cancer cells much less than clinical first-line drug, DDP. As PGPIPN and DDP together decreased the viabilities of drug-resistant ovarian cancer cells much more than these of sensitive cells, this suggested PGPIPN and DDP were synergistic effects, not additive effects. Our results also show that the peptide may increase the sensitivity of ovarian cancer cells to DDP by HSF1/HSP70 signaling pathway, but related mechanisms need further research.

There are four types of heat shock transcription factors (HSF1, 2, 3 and 4). Heat shock factor 1 (HSF1) is the main transcriptional regulator of heat shock response, which can protect organisms from various environmental and pathological stimuli 33. Recent studies have found that HSF1 may be related to tumorigenesis, and is activated in the process of canceration, and is closely related to the survival and proliferation of cancer cells 34. The expression of HSF1 in many tumor cells is immense, and ultraviolet radiation and thermal stimulation, etc. can activate the expression of HSF1 gene. HSFs can activate the expression of heat shock proteins (HSPs) in cells by sensing changes inside and outside the cell. HSF1 protects cancer cells from apoptosis and induces drug resistance by stimulating the transcription of genes encoding HSPs. This stimulatory action depends on the association of trimeric HSF1 to sequences within HSP gene promoter 35, 36. HSPs can regulate denatured proteins and structural proteins in cells through a series of reactions. By regulating a series of proteins, HSF1 and HSP can aggravate the deterioration of tumor cells, make tumor cells grow and proliferate, and promote the expression of tumor cell genes and proteins. There are many reports about the high expression of HSP70 in malignant tumors. And there are reports that after HSF1 is activated, it can regulate the expression of Hsp70 and induce carboplatin resistance in cancer cells 37.

Multidrug resistance (MDR) is considered to be one of the bottlenecks in the successful clinical treatment of many chemotherapy drugs. MDR1 (P-glycoprotein, P-gp) is a unique ATP-dependent membrane transporter and is one of the key regulators present in many tissues and organs 38, 39. MDR proteins have a variety of types, and MDR1 is a new type of protective barrier that prevents xenobiotin from entering the body. Some studies have showed that MDR1 could pump drugs out of the cell, which prevented cancer drugs from reaching their cellular targets, resulting in a reduction in the accumulation of many chemotherapeutic agents in the cell and interfering with the DNA replication pathway 40. The upregulation of p-gp is closely related to the increase of chemoresistance in cancer cells 41.The overexpression of MDR1 energized by ATP will help the development of MDR and ultimately reduce the effectiveness of chemotherapeutic agents. In addition to inducing HSPs to reduce chemotherapy-mediated cell killing, HSF1 also seems to be involved in multiple ways of regulating the expression of MDR1. It is known that the MDR1 gene contains heat shock elements (HSE) in the promoter region, and HSF1 can independently activate the expression of MDR1 42.

In recent years, there have been many studies on the direct relationship between ERCC1 and cisplatin resistance in ovarian cancer. Studies have shown that in patients who have received cisplatin chemotherapy drugs, ERCC1 is expressed at high doses 43. ERCC1 is one of the most important components of the nucleotide excision repair (NER) enzyme family; it is involved in the process of DNA excision and repair. The high expression of ERCC1 can repair damaged DNA molecules, increase the number of surviving cancer cells, and lead to tumor resistance to DDP 44. If the expression of ERCC1 is interfered, it will increase the sensitivity of chemotherapeutic drugs and thus achieve the purpose of treating tumors.

This experiment confirms that PGPIPN could increase the sensitivity of ovarian cancer cells to DDP in human ovarian cancer cell lines (SKOV3, COC1), their DDP-resistant cell lines (SKOV3/DDP, COC1/DDP) and human primary ovarian cancer cells. PGPIPN is a bioactive peptide derived from milk protein. Some bioactive peptides from bovine caseins could bind the opioid receptor on the cytomembrane to influence the proliferation, cytokine secretion, immune response and cell differentiation 45, 46. Recent studies have reported effects of bioactive peptides derived from milk proteins on the activities of toll-like receptor and human insulin receptor 47, 48. To gain insight into the mechanism by which PGPIPN increase the sensitivity of ovarian cancer cells to DDP, we used RNA-Seq technology to identify transcriptional alterations induced by PGPIPN in human primary ovarian cancer cell. We found HSF1 and HSP70, etc. were downregulated (data not shown). In this study, we validated the altered expression of HSF1, HSP70, MDR1 and ERCC1 genes by qPCR and western blotting. Therefore, it was speculated that PGPIPN may regulate HSF1/HSP70 signal transduction and alter the expression level or activity of proteins in human ovarian cancer cells, which leads to the reduction of MDR1 and ERCC1 expression, thereby increasing the sensitivity of ovarian cancer cells to DDP. However, this requires further investigation. Moreover, the present results provide new ideas for the development of ovarian cancer chemotherapy adjuvant drugs.

## Figures and Tables

**Table 1:** Sequences of the primers for real-time PCR

Gene	Primers (5′-3')
*HSF1*	Forward: CCATGAAGCATGAGAATGAGGC
	Reverse: CTTGTTGACGACTTTCTGTTGC
*HSP70*	Forward: ACCTACTCTTGTGTGGGTGTT
	Reverse: GACATAGCTTGGAGTGGTTCG
*MDR1*	Forward: GGGTTCTT CATGAATCTGG
	Reverse: CTGAATGTAAGCAGCAA CC
*ERCC1*	Forward: TGCCCATTCACTGCCTCCT
	Reverse: GCCTCGGTTCTGTGCCTTT
*β-actin*	Forward: GACACTTTCGAACACGTGATAG
	Reverse: TACAAGTCTGCGTCCTTATTGT

## References

[1] Wang W, Cao Y, Zhou X, Wei B, Zhang Y, Liu X (2018). PTP1B promotes the malignancy of ovarian cancer cells in a JNK-dependent mechanism. Biochem Biophys Res Commun.

[2] Dong X, Men X, Zhang W, Lei P (2014). Advances in tumor markers of ovarian cancer for early diagnosis. Indian J Cancer.

[3] Koshiyama M, Matsumura N, Konishi I (2017). Subtypes of Ovarian Cancer and Ovarian Cancer Screening. Diagnostics (Basel).

[4] Guan LY, Lu Y (2018). New developments in molecular targeted therapy of ovarian cancer. Discov Med.

[5] Barnett R (2016). Ovarian cancer. Lancet.

[6] Blagden SP, Nicum S (2021). A source of hope for platinum-resistant ovarian cancer?. Lancet.

[7] Christie EL, Bowtell DDL (2017). Acquired chemotherapy resistance in ovarian cancer. Ann Oncol.

[8] Diaz Osterman CJ, Ozmadenci D, Kleinschmidt EG, Taylor KN, Barrie AM, Jiang S (2019). FAK activity sustains intrinsic and acquired ovarian cancer resistance to platinum chemotherapy. Elife.

[9] Fekete JT, Osz A, Pete I, Nagy GR, Vereczkey I, Gyorffy B (2020). Predictive biomarkers of platinum and taxane resistance using the transcriptomic data of 1816 ovarian cancer patients. Gynecol Oncol.

[10] Qi X, Yu C, Wang Y, Lin Y, Shen B (2019). Network vulnerability-based and knowledge-guided identification of microRNA biomarkers indicating platinum resistance in high-grade serous ovarian cancer. Clin Transl Med.

[11] Xing F, Zhang L, Tang Z, Li X, Gong H, Wang B (2021). [Effect of thoraco-laparoscopic esophagectomy on postoperative immune function of patients with esophageal carcinoma]. Nan fang yi ke da xue xue bao = Journal of Southern Medical University.

[12] Gadducci A, Guarneri V, Peccatori FA, Ronzino G, Scandurra G, Zamagni C (2019). Current strategies for the targeted treatment of high-grade serous epithelial ovarian cancer and relevance of BRCA mutational status. J Ovarian Res.

[13] Pokhriyal R, Hariprasad R, Kumar L, Hariprasad G (2019). Chemotherapy Resistance in Advanced Ovarian Cancer Patients. Biomark Cancer.

[14] Wang Z, Li F, Wei M, Zhang S, Wang T (2020). Circadian Clock Protein PERIOD2 Suppresses the PI3K/Akt Pathway and Promotes Cisplatin Sensitivity in Ovarian Cancer. Cancer Manag Res.

[15] Yan W, Yang Y, Yang W (2019). Inhibition of SKP2 Activity Impaired ATM-Mediated DNA Repair and Enhanced Sensitivity of Cisplatin-Resistant Mantle Cell Lymphoma Cells. Cancer Biother Radiopharm.

[16] Huang WJ, Ruan S, Wen F, Lu XN, Gu SP, Chen XX (2020). Multidrug Resistance of Gastric Cancer: The Mechanisms and Chinese Medicine Reversal Agents. Cancer Manag Res.

[17] Mohanty DP, Mohapatra S, Misra S, Sahu PS (2016). Milk derived bioactive peptides and their impact on human health - A review. Saudi journal of biological sciences.

[18] Zhou J, Zhao M, Tang Y, Wang J, Wei C, Gu F (2016). The milk-derived fusion peptide, ACFP, suppresses the growth of primary human ovarian cancer cells by regulating apoptotic gene expression and signaling pathways. BMC Cancer.

[19] Aaghaz S, Gohel V, Kamal A (2019). Peptides as Potential Anticancer Agents. Current topics in medicinal chemistry.

[20] Li X, Wu H, Ouyang X, Zhang B, Su X (2017). New bioactive peptide reduces the toxicity of chemotherapy drugs and increases drug sensitivity. Oncol Rep.

[21] Meisel H (2005). Biochemical properties of peptides encrypted in bovine milk proteins. Curr Med Chem.

[22] Xu Q, Xi H, Chen X, Xu Y, Wang P, Li J (2020). Milk-derived hexapeptide PGPIPN prevents and attenuates acute alcoholic liver injury in mice by reducing endoplasmic reticulum stress. International journal of molecular medicine.

[23] Qi N, Liu C, Yang H, Shi W, Wang S, Zhou Y (2017). Therapeutic hexapeptide (PGPIPN) prevents and cures alcoholic fatty liver disease by affecting the expressions of genes related with lipid metabolism and oxidative stress. Oncotarget.

[24] Wang W, Gu F, Wei C, Tang Y, Zheng X, Ren M (2013). PGPIPN, a therapeutic hexapeptide, suppressed human ovarian cancer growth by targeting BCL2. PLoS One.

[25] Zhao M, Wei C, Yang X, Zhou J, Wang J, Gu F (2016). The milk-derived hexapeptide PGPIPN inhibits the invasion and migration of human ovarian cancer cells by regulating the expression of MTA1 and NM23H1 genes. Int J Oncol.

[26] Izaguirre DI, Ng CW, Kwan SY, Kun EH, Tsang YTM, Gershenson DM (2020). The Role of GDF15 in Regulating the Canonical Pathways of the Tumor Microenvironment in Wild-Type p53 Ovarian Tumor and Its Response to Chemotherapy. Cancers (Basel).

[27] Kazmierczak D, Jopek K, Sterzynska K, Ginter-Matuszewska B, Nowicki M, Rucinski M (2020). The Significance of MicroRNAs Expression in Regulation of Extracellular Matrix and Other Drug Resistant Genes in Drug Resistant Ovarian Cancer Cell Lines. Int J Mol Sci.

[28] Vaidyanathan A, Sawers L, Gannon AL, Chakravarty P, Scott AL, Bray SE (2016). ABCB1 (MDR1) induction defines a common resistance mechanism in paclitaxel- and olaparib-resistant ovarian cancer cells. Br J Cancer.

[29] Choi YE, Meghani K, Brault ME, Leclerc L, He YJ, Day TA (2016). Platinum and PARP Inhibitor Resistance Due to Overexpression of MicroRNA-622 in BRCA1-Mutant Ovarian Cancer. Cell Rep.

[30] Varghese E, Samuel SM, Liskova A, Samec M, Kubatka P, Busselberg D (2020). Targeting Glucose Metabolism to Overcome Resistance to Anticancer Chemotherapy in Breast Cancer. Cancers (Basel).

[31] Jin Z, Wang P, Chen J, He L, Xiao L, Yong K (2018). A Tumor-Specific Tissue-Penetrating Peptide Enhances the Efficacy of Chemotherapy Drugs in Gastric Cancer. Yonsei Med J.

[32] Wang F, Su H, Xu D, Dai W, Zhang W, Wang Z (2020). Tumour sensitization via the extended intratumoural release of a STING agonist and camptothecin from a self-assembled hydrogel. Nat Biomed Eng.

[33] Li N, Wang T, Li Z, Ye X, Deng B, Zhuo S (2019). Dorsomorphin induces cancer cell apoptosis and sensitizes cancer cells to HSP90 and proteasome inhibitors by reducing nuclear heat shock factor 1 levels. Cancer Biol Med.

[34] Chen L, Yang X (2019). TRIM11 cooperates with HSF1 to suppress the anti-tumor effect of proteotoxic stress drugs. Cell Cycle.

[35] Qu Z, Titus A, Xuan Z, D'Mello SR (2018). Neuroprotection by Heat Shock Factor-1 (HSF1) and Trimerization-Deficient Mutant Identifies Novel Alterations in Gene Expression. Sci Rep.

[36] Kim HB, Lee SH, Um JH, Oh WK, Kim DW, Kang CD (2015). Sensitization of multidrug-resistant human cancer cells to Hsp90 inhibitors by down-regulation of SIRT1. Oncotarget.

[37] Sharma A, Meena AS, Bhat MK (2010). Hyperthermia-associated carboplatin resistance: differential role of p53, HSF1 and Hsp70 in hepatoma cells. Cancer Sci.

[38] Muderrisoglu A, Babaoglu E, Korkmaz ET, Ongun MC, Karabulut E, Iskit AB (2020). Effects of Genetic Polymorphisms of Drug Transporter ABCB1 (MDR1) and Cytochrome P450 Enzymes CYP2A6, CYP2B6 on Nicotine Addiction and Smoking Cessation. Front Genet.

[39] Lai JI, Tseng YJ, Chen MH, Huang CF, Chang PM (2020). Clinical Perspective of FDA Approved Drugs With P-Glycoprotein Inhibition Activities for Potential Cancer Therapeutics. Front Oncol.

[40] Seelig A (2020). P-Glycoprotein: One Mechanism, Many Tasks and the Consequences for Pharmacotherapy of Cancers. Front Oncol.

[41] Nanayakkara AK, Follit CA, Chen G, Williams NS, Vogel PD, Wise JG (2018). Targeted inhibitors of P-glycoprotein increase chemotherapeutic-induced mortality of multidrug resistant tumor cells. Sci Rep.

[42] Vilaboa NE, Galan A, Troyano A, de Blas E, Aller P (2000). Regulation of multidrug resistance 1 (MDR1)/P-glycoprotein gene expression and activity by heat-shock transcription factor 1 (HSF1). J Biol Chem.

[43] Zhang Z, Dou X, Yang H, Jia L, Qin K, Gao X (2020). Association of expression of p53, livin, ERCC1, BRCA1 and PARP1 in epithelial ovarian cancer tissue with drug resistance and prognosis. Pathol Res Pract.

[44] Xia A, Li H, Li R, Lu L, Wu X (2018). Co-treatment with BEZ235 enhances chemosensitivity of A549/DDP cells to cisplatin via inhibition of PI3K/Akt/mTOR signaling and downregulation of ERCC1 expression. Oncol Rep.

[45] Fiedorowicz E, Jarmolowska B, Iwan M, Kostyra E, Obuchowicz R, Obuchowicz M (2011). The influence of mu-opioid receptor agonist and antagonist peptides on peripheral blood mononuclear cells (PBMCs). Peptides.

[46] Ledesma-Martínez E, Aguíñiga-Sánchez I, Weiss-Steider B, Rivera-Martínez AR, Santiago-Osorio E (2019). Casein and Peptides Derived from Casein as Antileukaemic Agents. Journal of oncology.

[47] Ashraf A, Mudgil P, Palakkott A, Iratni R, Gan CY, Maqsood S (2021). Molecular basis of the anti-diabetic properties of camel milk through profiling of its bioactive peptides on dipeptidyl peptidase IV (DPP-IV) and insulin receptor activity. Journal of dairy science.

[48] Drago-Serrano ME, Campos-Rodriguez R, Carrero JC, de la Garza M (2018). Lactoferrin and Peptide-derivatives: Antimicrobial Agents with Potential Use in Nonspecific Immunity Modulation. Current pharmaceutical design.

